# An integrative analysis of ASCL1 in breast cancer and inhibition of ASCL1 increases paclitaxel sensitivity by activating ferroptosis via the CREB1/GPX4 axis

**DOI:** 10.3389/fimmu.2025.1546794

**Published:** 2025-02-03

**Authors:** Xiaolu Yang, Yilun Li, Yaqi Peng, Yuan Chang, Binglu He, Tianqi Zhang, Shiyu Zhang, Cuizhi Geng, Yunjiang Liu, Xiaolong Li, Jun Hao, Li Ma

**Affiliations:** ^1^ Department of Breast Disease Center, The Fourth Hospital of Hebei Medical University, Shijiazhuang, China; ^2^ Department of Pathology, Hebei Medical University, Shijiazhuang, China; ^3^ Department of Breast Disease Center, Affiliated Hospital of Hebei University of Engineering, Handan, China; ^4^ Department of Breast Disease Center, Xingtai Renmin Hospital, Xingtai, China; ^5^ Department of Breast Disease Center, The Fourth Hospital of Shijiazhuang, Shijiazhuang, China

**Keywords:** breast cancer, ASCL1, ferroptosis, therapeutic target, drug sensitivity

## Abstract

**Objective:**

Our previous study found that Achaete-scute complex homolog 1 (ASCL1) is involved in classifying BC subtypes with different prognostic and pathological characteristics. However, the biological role of ASCL1 in BC still remains largely unexplored. This study aims to elucidate the function of ASCL1 in BC using bioinformatics analyses, as well as *in vitro* and *in vivo* experimental approaches.

**Methods:**

Data from the TCGA, GEO, and Human Protein Atlas databases were utilized to evaluate ASCL1 expression in BC and its association with patient prognosis. Genetic alterations in ASCL1 were assessed through the COSMIC and cBioPortal databases, while the TIMER2.0 database provided insights into the relationship between ASCL1 expression and key gene mutations in BC. The GDSC database was used to examine correlations between ASCL1 levels and sensitivity to standard chemotherapeutic agents. Associations between ASCL1 expression and cytokines, immunomodulatory factors, MHC molecules, and receptors were analyzed using Pearson and Spearman correlation methods. The TIP database was employed to investigate the connection between ASCL1 expression and immunoreactivity scores, and six computational approaches were applied to evaluate immune cell infiltration. Functional assays were conducted on BC cell lines MCF-7 and MDA-MB-231, and nude mouse models were used for *in vivo* studies.

**Results:**

ASCL1 was found to be upregulated in BC and correlated with unfavorable prognosis and mutations in key oncogenes. Its expression was linked to immunomodulatory factors, immune cell infiltration, and immunoreactivity scores in the tumor microenvironment. Additionally, ASCL1 influenced tumor immune dynamics and chemosensitivity in BC. Overexpression of ASCL1 enhanced BC cell proliferation, migration and invasion, while its knockdown had the opposite effect. Notably, inhibition of ASCL1 increased BC cell sensitivity to paclitaxel both *in vitro* and *in vivo*. In addition, inhibition of ASCL1 activated ferroptosis in BC, including altered mitochondrial morphology, increased MDA and ROS levels, decreased GSH levels and reduced GSH/GSSG ratio. Mechanistically, inhibition of ASCL1 decreases the phosphorylation of CREB1, thus reducing the expression of GPX4. In summary, inhibition of ASCL1 increases paclitaxel sensitivity by activating ferroptosis via the CREB1/GPX4 axis.

**Conclusions:**

ASCL1 exerts oncogenic effects in BC and represents a potential therapeutic target for intervention.

## 1 Introduction

Breast cancer is the most prevalent malignant tumor among women worldwide (1), with 2,308,897 new cases in 2022, and its incidence continues to rise annually (1, 2). Although a variety of therapeutic options are available—such as surgery, chemotherapy, endocrine therapy, radiotherapy, immunotherapy and anti-HER2 therapy—BC is still the leading cause of cancer-related deaths among women (1, 3). This underscores the urgent need for advancements that can enhance patient survival.

Molecular markers in cancer serve as measurable indicators at the molecular level, aiding in accurate diagnosis, risk assessment, treatment response prediction, and prognosis evaluation. Moreover, many of the new targeted therapies are effective only in patients harboring specific genetic mutations, making molecular biomarkers essential for identifying these patient subgroups (4). Consequently, the discovery of novel BC tumor markers with high specificity and sensitivity is essential for improving patient outcomes.

ASCL1, a member of the basic helix-loop-helix (bHLH) family of transcription factors (5), was initially identified in the 1980s in Drosophila melanogaster as a proneural gene due to its ability to induce neural identity in naïve ectodermal cells (6). As a key bHLH family member, ASCL1 regulates glial cell proliferation and specifies neuronal phenotype and subtype during forebrain development (7–10). This led to its early association with nervous system development. However, in 2014, ASCL1 was recognized as an oncogene and therapeutic target in high-grade neuroendocrine lung cancer (11). Subsequent research has increasingly linked ASCL1 overexpression to oncogenic activity in various cancers, with significant associations with poor prognosis and therapeutic resistance. For example, Miyashita N et al. (12, 13) reported that in lung adenocarcinoma, patients with ASCL1 high expression had an attenuated response to immunotherapy. Similarly, Wei J et al. (14) found that among individuals with small cell lung cancer (SCLC), those who were ASCL1-positive had significantly poorer survival outcomes compared to ASCL1-negative patients. Furthermore, ASCL1 overexpression facilitates prostate cancer progression and metastasis, while its inhibition reduces tumor cell proliferation (15). These findings consistently demonstrate ASCL1’s role in promoting tumor progression.

In addition to promoting the progression of a wide range of tumors, ASCL1 has been recognized as a molecular marker for delineating SCLC subtypes. In 2019, Rudin et al. (16) proposed to classify SCLC into four subtypes, A, N, Y, and P, based on four transcription factors (ASCL1, NeuroD1, YAP1, and POU2F3), advancing the development of precision therapy for SCLC. Currently, this classification criterion has been widely used in SCLC. In addition, a previous study by our team also demonstrated that ASCL1 is an important chromatin regulator that helps to classify BC subtypes with different prognostic and pathological features (17). This finding suggests a potential role for ASCL1 in BC. However, the biological role of ASCL1 in BC still remains largely unexplored. Therefore, we conducted this study.

This study aims to systematically evaluate the multifaceted role of ASCL1 in BC, focusing on its influence on biological processes, tumor immunology, treatment response, and prognosis, using a combination of bioinformatics, *in vitro*, and *in vivo* methodologies. Additionally, this study elucidates the specific molecular mechanisms through which ASCL1 regulates BC progression and assesses its potential as a novel therapeutic target.

## 2 Methods

### 2.1 Collection of data

RNA-seq data of ASCL1 for 33 pan-cancers and normal breast tissues were obtained from the Cancer Genome Atlas (TCGA) and the Genotype-Tissue Expression Project (GTEx) database, respectively. Relationship between ASCL1 mRNA expression and clinical survival data were sourced from the Gene Expression Omnibus (GEO) database (GSE11121, GSE20711). Additionally, immunohistochemical staining data for ASCL1 in normal breast tissue and BC samples were collected from the Human Protein Atlas (HPA) database and the Fourth Hospital of Hebei Medical University, respectively. Further details on other databases utilized in this study are provided below.

### 2.2 Differential expression analysis

To analyze ASCL1 mRNA expression, comparisons were made between normal and tumor tissues. Duplicate and missing RNA-seq entries from the GTEx and TCGA databases were excluded, and the remaining data were log-transformed to log_2_(TPM + 1). Western blot analysis was used to evaluate the protein expression levels of ASCL1, comparing the human normal breast epithelial cell line (MCF-10A) to BC cell lines (MDA-MB-231 and MCF-7). Immunohistochemical staining further evaluated ASCL1 expression intensity in normal breast and BC tissues.

### 2.3 Survival analysis

Based on the median ASCL1 mRNA level, patients were divided into a high ASCL1 expression group and a low ASCL1 expression group for subsequent analyses. Prognostic associations between ASCL1 expression and outcomes of patients with BC, including recurrence-free survival (RFS) and overall survival (OS), were investigated.

### 2.4 Genetic alteration analysis

Mutation frequency and specific mutation sites of ASCL1 in BC were examined using the cBioPortal database (18), while the Catalogue of Somatic Mutations in Cancer (COSMIC) database was employed to categorize mutation types in ASCL1 in BC (19).

Additionally, the relationship between the ASCL1 expression and mutations in key BC-related genes was also evaluated using a chi-square test. All mutation data of TCGA samples were derived from the Genomic Data Commons (GDC) database (20).

### 2.5 Immunomodulatory analysis

Correlation analyses between ASCL1 expression and immunomodulatory factors—including cytokines, immunoregulators, MHC molecules, and receptors—were performed across pan-cancer datasets using Pearson and Spearman methods. The relevant data were acquired from the TCGA database.

### 2.6 Immune activity score analysis

The Tracking Tumor Immunophenotype (TIP) database was employed to assess the relationship between ASCL1 expression and immune activity scores. The TIP platform allowed systematic analysis and visualization of the proportions of tumor-infiltrating immune cells across the seven-step Cancer-Immunity Cycle (21).

### 2.7 Immune cell infiltration analysis

Data on immune cell infiltration were retrieved from the TIMER2.0 database, and immune cell infiltration scores were calculated to analyze their correlation with ASCL1 expression using six methods: TIMER, EPIC, XCELL, CIBERSORT, QUANTISEQ, and MCP-COUNTER (22).

### 2.8 Drug sensitivity analysis

To explore the relationship between ASCL1 expression and sensitivity to common chemotherapeutic agents used in BC treatment. RNAseq data and corresponding clinical information for BC were obtained from the TCGA database. Subsequently, the chemotherapy response of each sample was predicted based on the Genomics of Drug Sensitivity in Cancer (GDSC) database. The prediction process was implemented using the pRRophetic package of R software, and the half-maximal inhibitory concentration (IC50) of the samples was estimated using ridge regression. All parameters were set as the default values. Using the batch effect of combat and tissue type of all tissues, the duplicate gene expression was summarized as a mean value.

### 2.9 Pathway enrichment analysis

To investigate the pathways by which ASCL1 regulates BC progression, Gene Set Enrichment Analysis (GSEA) and Kyoto Encyclopedia of Genes and Genomes (KEGG) pathway analyses were employed to elucidate the differential gene enrichment between high ASCL1 and low ASCL1 expression groups in BC.

### 2.10 Cell culture

The three cell lines were obtained from the Cell Resource Centre of the Chinese Academy of Medical Sciences. All these cells were cultured using the DMEM medium containing 4.5 g glucose (Gibco, USA). The medium contained 10% fetal bovine serum (FBS) and 1% penicillin/streptomycin (Gibco, USA).

### 2.11 Lentivirus infection and cell transfection

To generate stable ASCL1 knockdown or overexpression in BC cells, lentivirus-mediated infection was performed. Plasmids and lentiviruses were transfected into HEK-293T cells, followed by infection of BC cells with virus-packed plasmids. Stable cell lines were selected using puromycin.

For cell transfection, The two BC cells were seeded in six-well plates. When the cells reached 70%-80% confluence, Lipofectamine 3000 and plasmids were used to transfect the cells. The efficiency of transfection was verified by qRT-PCR and Western blot analysis.

### 2.12 Cell viability assay

In this study, the CCK-8 assay (Yeasen, China) was utilized to assess cell proliferation and identify the IC50 of paclitaxel. For proliferation analysis, cells (2 × 10^3^) were seeded in the 96-well plates. CCK-8 reagent (10 μl) was added to each well on days 1, 2 and 3, respectively, and incubated for 2 h. The absorbance was then measured at 450 nm and the value of optical density (OD) 450 was recorded.

For IC50 determination, cells (5 × 10^3^) were seeded in 96-well plates and cultured for 24 hours. Afterwards, the cells were exposed to a paclitaxel-containing medium with varying concentrations. The tested concentrations for MCF-7 cells were 0, 0.01, 0.05, 0.1, 0.2, 0.5, and 1 mg/L, while those for MDA-MB-231 cells were 0, 0.1, 0.5, 1, 2, 4, and 8 mg/L. Cells were incubated for an additional 48 hours and then the OD450 of each well was measured.

To evaluate the association between BC sensitivity to paclitaxel and ferroptosis, cells (5 × 10^3^) were seeded in 96-well plates and cultured for 24 hours. Following this, drugs were added to the culture medium, with final concentrations of Erastin at 5 μM and ferrostatin-1 at 2 μM. Paclitaxel concentrations used were 0.01 mg/L for MCF-7 and 1 mg/L for MDA-MB-231. Cells were cultured for an additional 24 hours, and OD450 was measured.

### 2.13 Clone formation

Cells (1000 cells/well) were seeded in 6-well plates. After 14 days of culture, cells were fixed with 4% paraformaldehyde for 25 minutes, stained with 1% violet crystal for 20 minutes and colonies were counted.

### 2.14 Wound healing assay

For wound healing assays, cells were transfected and cultured using a 6-well plate then a 1 ml pipette tip was used to create a scratch, and wound closure was observed and photographed at 0, 24, and 48 hours using the microscope at 4x magnification.

### 2.15 Transwell assay

The Transwell assay was performed to assess the migration and invasive capacity of BC cells. For migration assays, 50,000 cells were inoculated in the upper chamber with DMEM medium without FBS. 650 μl DMEM medium containing FBS was added to the lower chamber. After 2 days, the cells on the bottom of the upper chamber were fixed with 4% formaldehyde for 25 minutes, stained with 1% crystal violet for 20 minutes, and then photographed at 10x magnification.

For invasion assays, the matrix (Beyotime, China) was diluted at a 1:7 ratio with FBS-free DMEM, and 60 µl was added to the upper chamber. After a 4-hour incubation to solidify the matrix gel, the remaining steps were identical to the migration assay.

### 2.16 Detection of ferroptosis indicators

The malondialdehyde (MDA) kit (BOXBIO, China), the reactive oxygen species (ROS) detection kit (Report Biotech, China) and the glutathione (GSH) and oxidized glutathione (GSSG) assay kit (Beyotime, China) were used to detect MDA level, ROS level, GSH level and GSH/GSSG ratio in cells following the manufacturer’s protocol.

### 2.17 Quantitative reverse transcription PCR

Total RNA was extracted from BC cells and then synthesized into cDNA and performed qRT-PCR. The quantification of target gene expression was calculated using the 2^−ΔΔCt^ method based on cycle threshold (Ct) values.

### 2.18 Western blot analysis

Protein samples (40 µg each) extracted from the cells were first separated by electrophoresis on 10% SDS-PAGE. The proteins were then transferred to a PVDF membrane and blocked with 5% skimmed milk. The membrane was then incubated with primary antibody for 8-12 hours at 4°C and washed 3 times with PBST, then incubated with secondary antibody for 2 hours and washed 3 times with PBST.

### 2.19 Co-immunoprecipitation

The rProtein A/G Magnetic IP/Co-IP Kit was used for Co-IP (ACE biotechnology, China). Cell lysates were first incubated with antibodies and magnetic beads at 4°C overnight. Then discard the supernatant and rinse the beads. Finally, the magnetic beads were eluted and subjected to western blotting.

### 2.20 *In vivo* study

For *in vivo* experiments, female BALB/c nude mice (Vital River, China) were used. Stably knocked-down sh-ASCL1-MCF-7 cells and control sh-NC-MCF-7 cells (1 × 10^7^ cells) were injected subcutaneously in the right axillary region of the mice. One week post-injection, paclitaxel was administered intraperitoneally at a dose of 1 mg/kg every three days. After three weeks, the mice were euthanized, and subcutaneous tumors were excised to measure their weight. In addition, tumor volume was measured every five days. The formula is tumor volume = π/6 × length × width^2^.

### 2.21 Statistical analysis

GraphPad Prism 9.5 and R software were used for the statistical analyses. The t-test, Wilcoxon test, and two-way ANOVA were used to evaluate relationships between variables, and the log-rank test was applied to assess cancer prognosis. *P* < 0.05 was considered statistically significant.

## 3 Results

### 3.1 ASCL1 is highly expressed in BC

**Figure 1:**
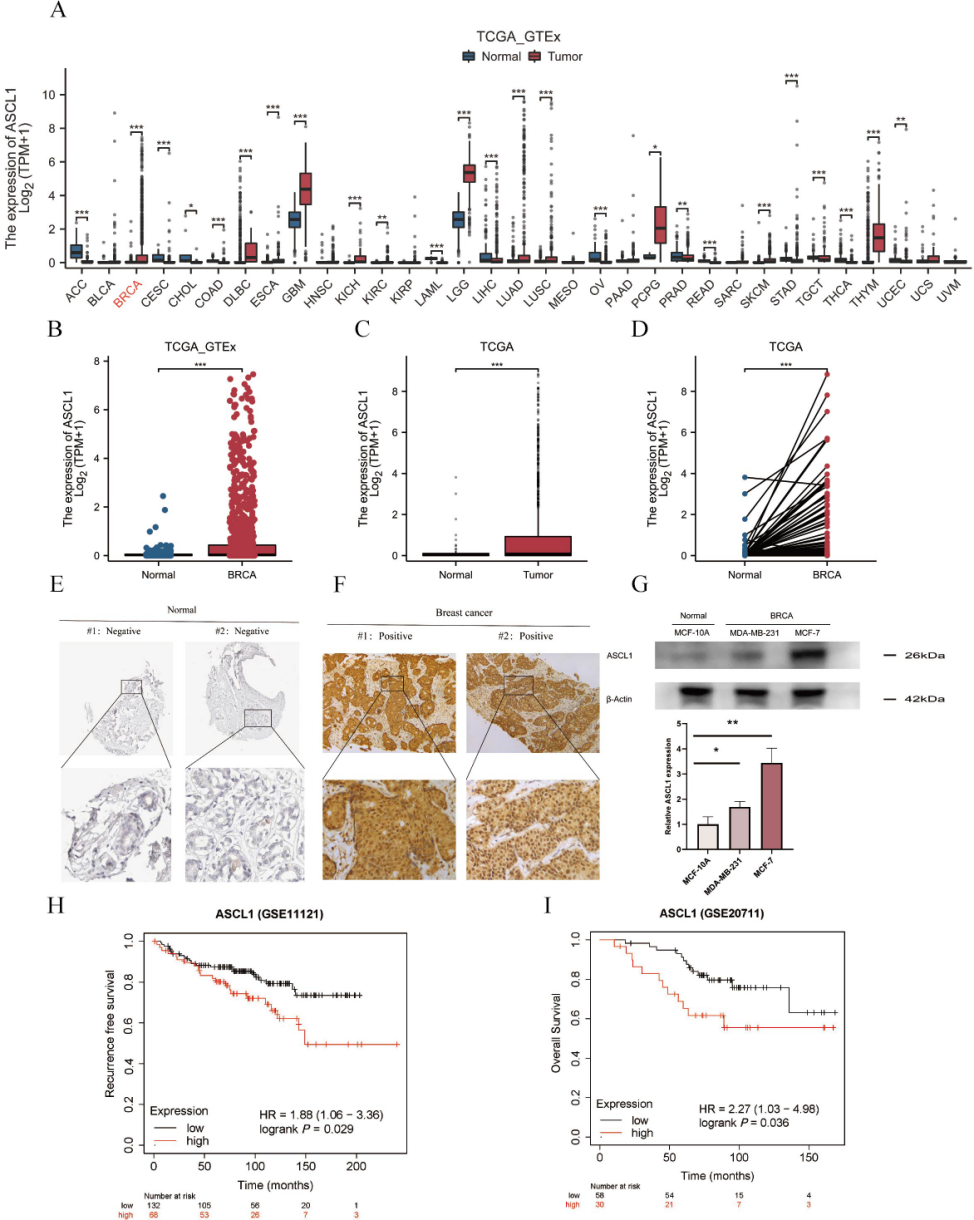
ASCL1 is highly expressed in BC and correlates with poor prognosis. **(A)** Comparative analysis of ASCL1 mRNA expression across pan-cancers, including BC, and normal tissues based on data from the TCGA and GTEx databases. **(B, C)** ASCL1 mRNA expression comparison between BC and normal tissues using data from **(B)** the combined TCGA and GTEx databases, and **(C)** the TCGA database alone. **(D)** ASCL1 mRNA expression in paired BC and adjacent normal tissues from the TCGA database. **(E)** Immunohistochemical staining of ASCL1 in normal breast tissue from the HPA database. **(F)** Immunohistochemical staining of ASCL1 in BC tissues. **(G)** Protein expression levels of ASCL1 in normal breast epithelial cells and BC cell lines. **(H, I)** Prognostic impact of ASCL1 expression on **(H)** RFS, **(I)** OS in patients with BC. (*P < 0.05; **P < 0.01; ***P < 0.001).

The mRNA expression of ASCL1 was analyzed across multiple cancer types using combined data from the TCGA and GTEx databases, addressing the lack of normal individual data in TCGA. ASCL1 expression was significantly elevated in various cancer tissues, including BC (**Figures 1A, B**). Consistent results were obtained when analyzing TCGA data alone, showing increased ASCL1 expression in BC tissues compared to normal tissues, as confirmed by pairwise comparisons (**Figures 1C, D**).

Further investigation into ASCL1 protein levels revealed a similar trend. IHC data from the HPA database showed that ASCL1 was typically undetectable in normal breast tissue (**Figure 1E**), while strong positivity was observed in BC samples (**Figure 1F**). Western blot analysis further supported these results, indicating significantly higher ASCL1 protein levels in BC cells than in the normal breast epithelial cells (**Figure 1G**). These results consistently suggest that ASCL1 is overexpressed in BC.

### 3.2 ASCL1 is associated with poor prognosis in BC

The relationship between ASCL1 expression and prognosis in BC was also evaluated. Patients with elevated ASCL1 expression had significantly poorer RFS and OS compared to those with lower expression levels (**Figures 1H, I**). All these results imply that ASCL1 may play a cancer-promoting role in BC.

### 3.3 Genetic alterations of ASCL1 in BC

**Figure 2:**
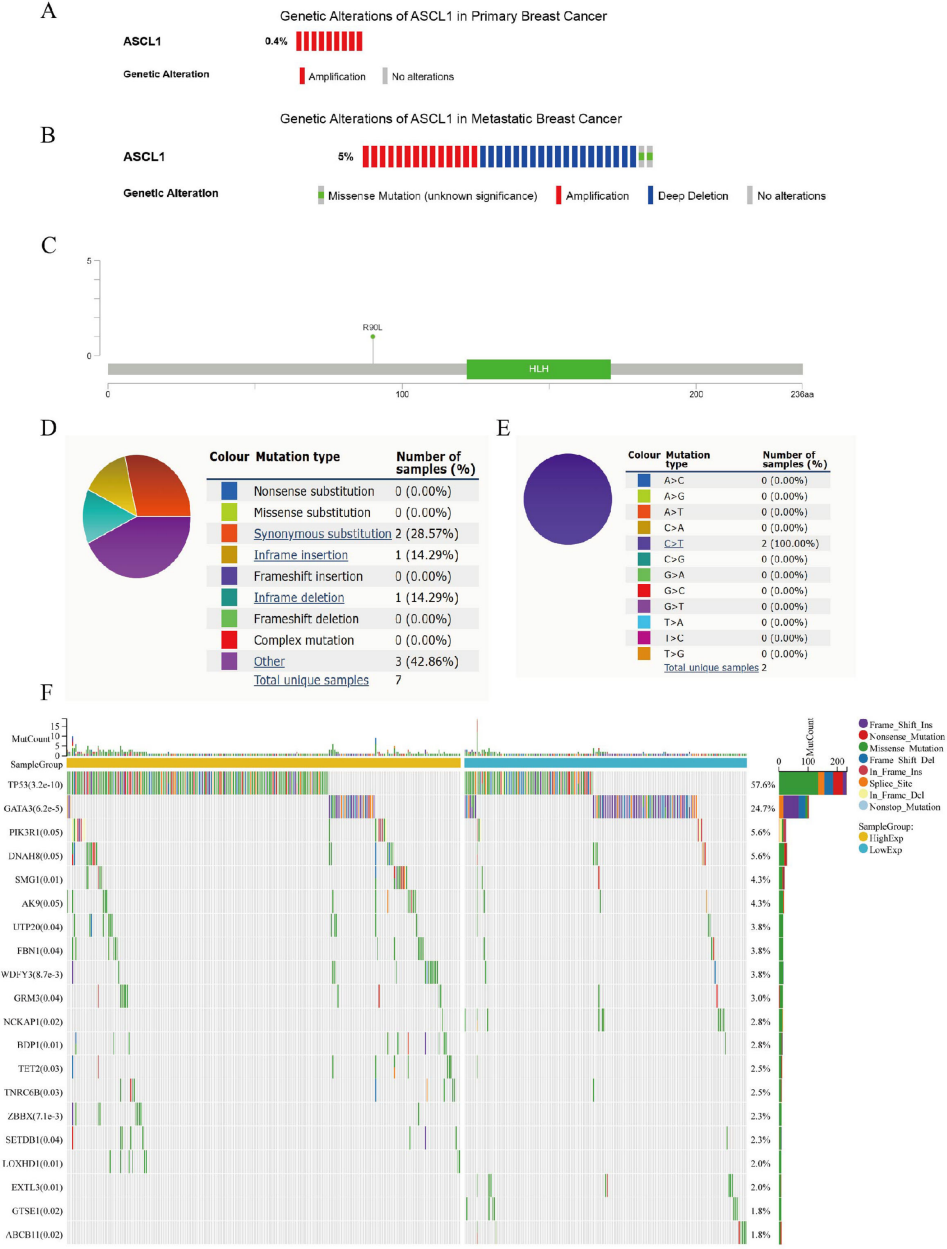
Genetic alterations of ASCL1 in BC and their relationship to mutations in other key genes. **(A, B)** Oncoprint representation of ASCL1 genetic alterations in **(A)** primary BC and **(B)** metastatic BC, based on data from the cBioPortal database. **(C)** Diagram showing ASCL1 mutations across protein domains in metastatic BC, derived from cBioPortal data. **(D, E)** Types of **(D)** ASCL1 mutations and **(E)** specific substitution mutation of ASCL1 in BC from the COSMIC database. **(F)** Mutation landscape of key genes stratified by high and low ASCL1 expression in BC.

The frequency and types of ASCL1 mutations in primary breast cancer were examined using a dataset of 2051 samples with complete DNA sequencing data from the cBioPortal database (23, 24). Additionally, two metastatic BC datasets, including 481 patients from The Metastatic Breast Cancer Project and 216 patients from Lefebvre C et al. (25), totaling 697 patients, were analyzed for ASCL1 alteration types and frequencies in metastatic cases. In primary BC, the ASCL1 alteration frequency was found to be 0.4% (9/2051) (**Figure 2A**). In contrast, the frequency in metastatic BC was significantly higher at 5% (35/697), consisting of 14 cases of amplification, 19 cases of deep deletion, and 2 cases of missense mutations, with a statistically significant difference between primary and metastatic groups (*P* < 0.001) (**Figure 2B**). Mutation mapping further identified specific sites of missense mutations in ASCL1 in metastatic cases (**Figure 2C**). In addition, we further analyzed the mutation frequency and type of ASCL1 in BC using the COSMIC database. Among 2779 BC samples that were tested, 7 exhibited ASCL1 mutations. These included 2 synonymous substitutions, 1 in-frame insertion, 1 in-frame deletion, and 3 other mutation types (**Figure 2D**). Both synonymous substitutions involved C>T transitions (**Figure 2E**).

### 3.4 ASCL1 correlates with mutations in major key genes in BC

The gene mutation landscape was compared between the high- and low-ASCL1 expression groups in BC (**Figure 2F**). Significant differences were observed in the mutation rates of several key genes, including TP53, GATA3, PI3KR1, SMG1, and UTP20. Notably, the mutation rates of TP53 and PIK3R1—genes strongly linked to poor prognosis in BC—were significantly higher in the ASCL1-high group, suggesting a potential association between elevated ASCL1 expression and BC progression.

### 3.5 ASCL1 is associated with immunomodulatory factors, immune cell infiltration, and immune activity score in BC

**Figure 3:**
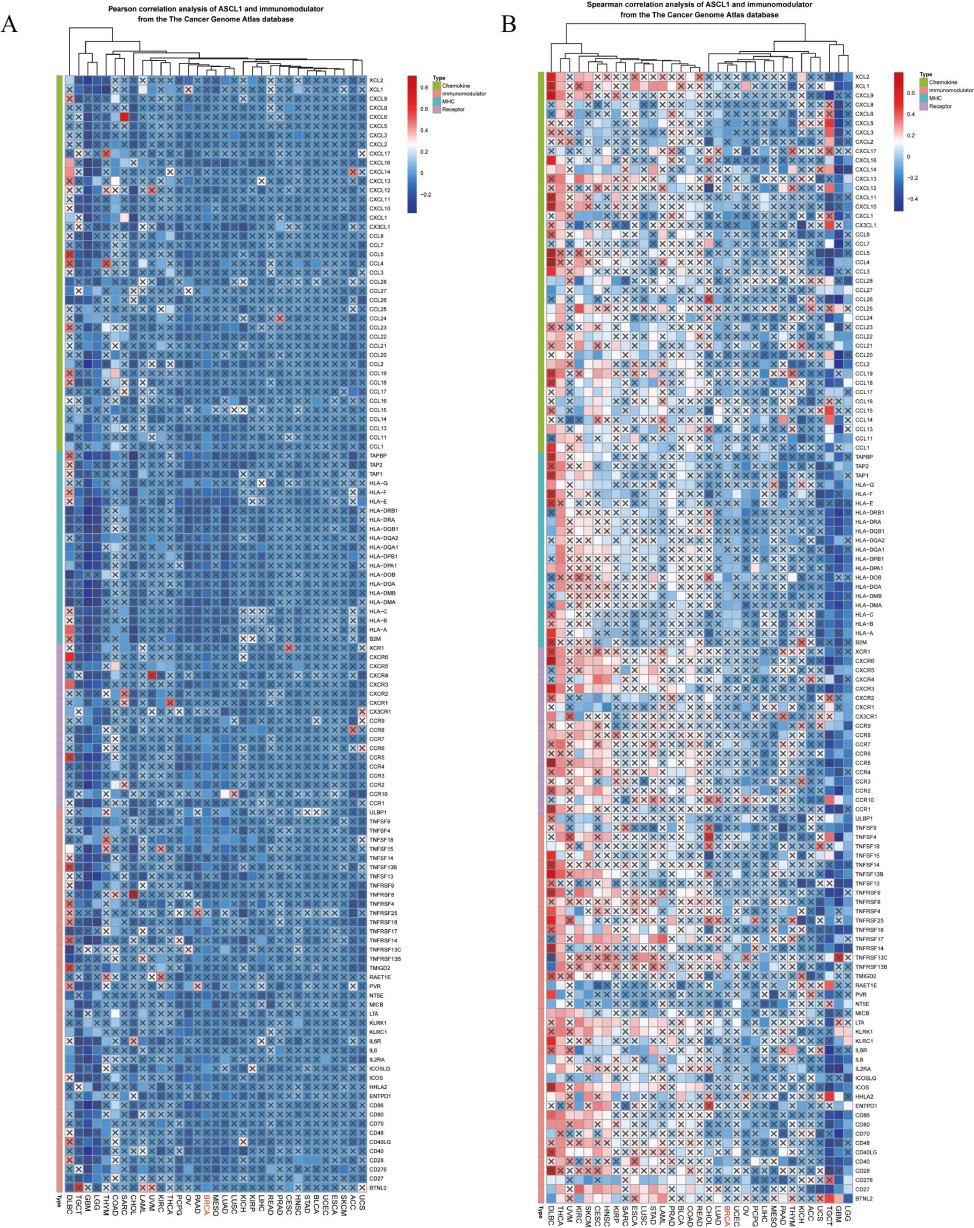
Correlation between ASCL1 expression and immunomodulatory factors in pan-cancer analysis. **(A)** Pearson and **(B)** Spearman correlation analyses between ASCL1 and immunomodulatory factors from the TCGA database.

To investigate the impact of elevated ASCL1 expression on BC, the relationship between ASCL1 and tumor immunity was examined. Given the critical role of the tumor microenvironment in modulating immune responses (26), ASCL1’s association with various immunomodulatory factors—including cytokines, immunomodulators, MHC molecules, and receptors—was assessed across multiple cancers, including BC. Both Pearson and Spearman correlation analyses demonstrated a strong association between ASCL1 and immunomodulatory factors in pan-cancer contexts (**Figures 3A, B**). In BC specifically, ASCL1 expression was negatively correlated with these factors.

**Figure 4:**
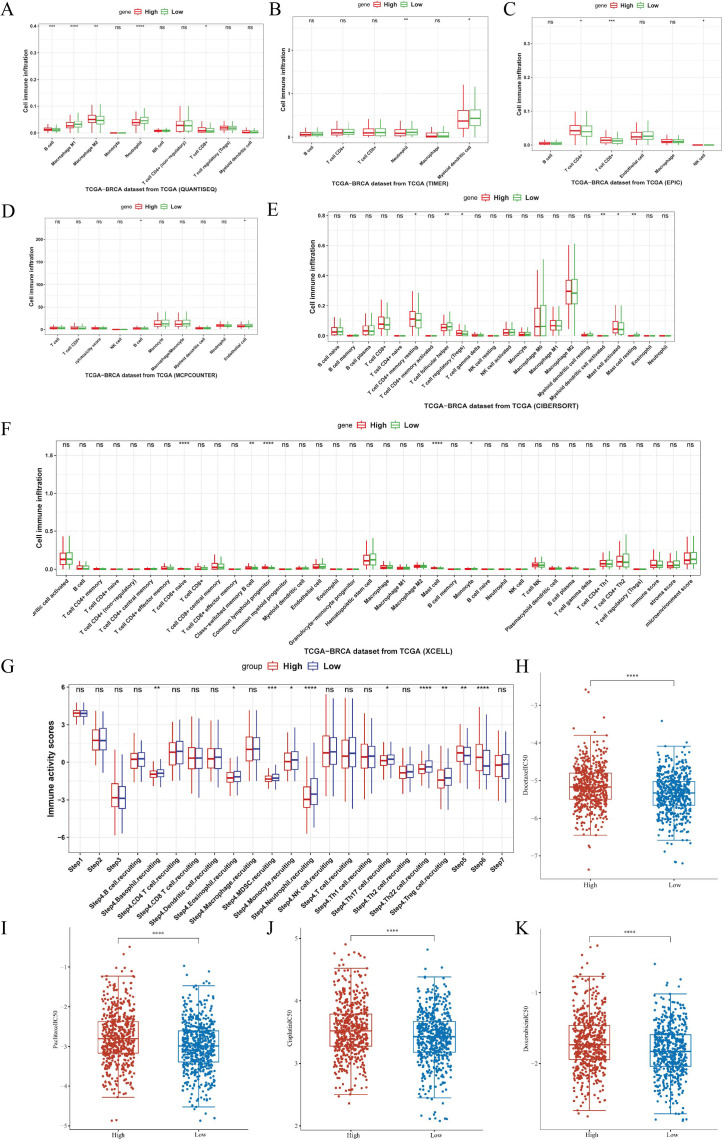
Association of ASCL1 expression with tumor immunity and chemotherapy response in BC. **(A-F)** Correlations between ASCL1 expression and immune cell infiltration, using various methods: **(A)** QUANTISEQ, **(B)** TIMER, **(C)** EPIC, **(D)** MCP-COUNTER, **(E)** CIBERSORT, and **(F)** XCELL. **(G)** Correlation between ASCL1 expression and immune activity score in BC. **(H-K)** Comparison of IC50 values for common chemotherapeutic drugs between high and low ASCL1 expression groups in BC: **(H)** docetaxel, **(I)** paclitaxel, **(J)** cisplatin, and **(K)** doxorubicin. (*P < 0.05; **P < 0.01; ***P < 0.001; ****P < 0.0001), ns, not significant.

The relationship between ASCL1 expression and immune cell infiltration was further explored using six different analytical methods, categorizing patients into ASCL1-high and ASCL1-low expression groups. ASCL1 expression showed associations with a wide range of cell types, including B cells, Treg cells, NK cells, CD4^+^ and CD8^+^ T cells, M1 and M2 macrophages, myeloid dendritic cells, neutrophils, monocytes, endothelial cells, and mast cells (**Figures 4A-F**). Notably, the ASCL1-high group exhibited increased M2 macrophage infiltration and decreased M1 macrophage infiltration. Given that M2 macrophages are known to promote tumor progression and suppress immune function in malignancies (27–29) (**Figure 4A**), these results indicate a potential role for ASCL1 in immune evasion. However, we also found that the effects of ASCL1 on the immune response were not entirely consistent, for example, patients in the high ASCL1 group had less neutrophil infiltration and more infiltration of Treg cells, CD4^+^ and CD8^+^ T cells. Normally neutrophils and Treg cells are considered to be pro-tumor progression cells, while CD4^+^ and CD8^+^ T cells are cells that inhibit tumor progression. These results seem to demonstrate that there are conflicting roles for the ASCL1 tumor immune response, but what is certain is that ASCL1 is associated with the infiltration of immune cells in BC. We also assessed the relationship between ASCL1 expression and immunoreactivity scores, revealing that ASCL1 expression was also associated with immune response scores in BC (**Figure 4G**).

### 3.6 ASCL1 correlates with drug sensitivity in BC

Building on these results, the relationship between ASCL1 expression and sensitivity to chemotherapeutic drugs was evaluated using the GDSC database. Differences in the IC50 values of commonly used chemotherapeutics, including docetaxel, paclitaxel, cisplatin, and doxorubicin, were compared between the high- and low-ASCL1 expression groups. The IC50 values for all four drugs were significantly higher in the ASCL1-high group (**Figures 4H-K**), indicating reduced sensitivity to these treatments. This suggests that elevated ASCL1 expression negatively impacts chemotherapy efficacy in BC.

### 3.7 Inhibition of ASCL1 inhibits BC progression *in vitro*

**Figure 5:**
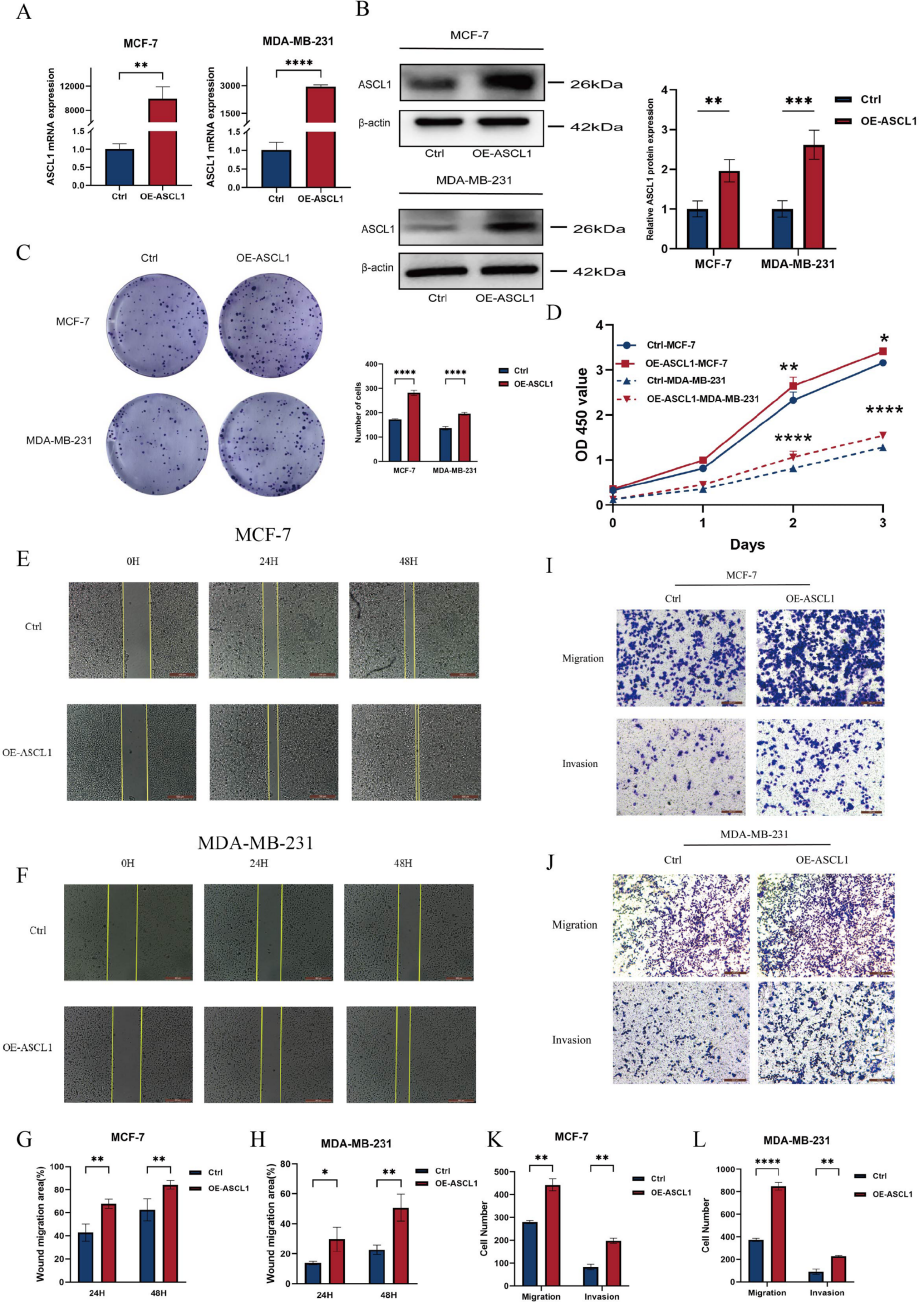
ASCL1 promotes BC cell progression. **(A, B)** Assessment of ASCL1 overexpression efficiency through **(A)** qRT-PCR and **(B)** Western blot analysis. **(C, D) (C)** Colony formation assay and **(D)** CCK-8 assay showing that ASCL1 overexpression enhances the proliferation of MCF-7 and MDA-MB-231 cells. **(E, F)** Wound healing assay demonstrating enhanced migration of **(E)** MCF-7 and **(F)** MDA-MB-231 cells with ASCL1 overexpression (scale bar = 500μm). **(G, H)** Histogram of percentage of wound migration area for **(G)** MCF-7 and **(H)** MDA-MB-231 cells. **(I, J)** Transwell assay revealing that ASCL1 overexpression promotes migration and invasion of **(I)** MCF-7 and **(J)** MDA-MB-231 cells (scale bar = 200μm). **(K, L)** Histogram of the results of transwell assay on **(K)** MCF-7 and **(L)** MDA-MB-231 cells. (*P < 0.05; **P < 0.01; ***P < 0.001; ****P < 0.0001).

The bioinformatics findings suggested a critical role for ASCL1 in BC progression, prompting further experimental validation. To investigate this, BC cell lines stably overexpressing ASCL1 (OE-ASCL1-MCF-7, OE-ASCL1-MDA-MB-231) were established. Both qRT-PCR and Western blot analyses confirmed that ASCL1 mRNA and protein levels were significantly increased in overexpressing cells (**Figures 5A, B**). In terms of functional tests, CCK-8 and colony formation tests showed that ASCL1 overexpression significantly increased the proliferation of BC cells (**Figures 5C, D**). The wound healing assay demonstrated a marked increase in cell migration (**Figures 5E-H**), which was further corroborated by Transwell assays showing that ASCL1 significantly promoted both migration and invasion (**Figures 5I-L**). These results collectively demonstrate that ASCL1 drives malignant behaviors in BC cells *in vitro*, promoting proliferation, migration and invasion.

**Figure 6:**
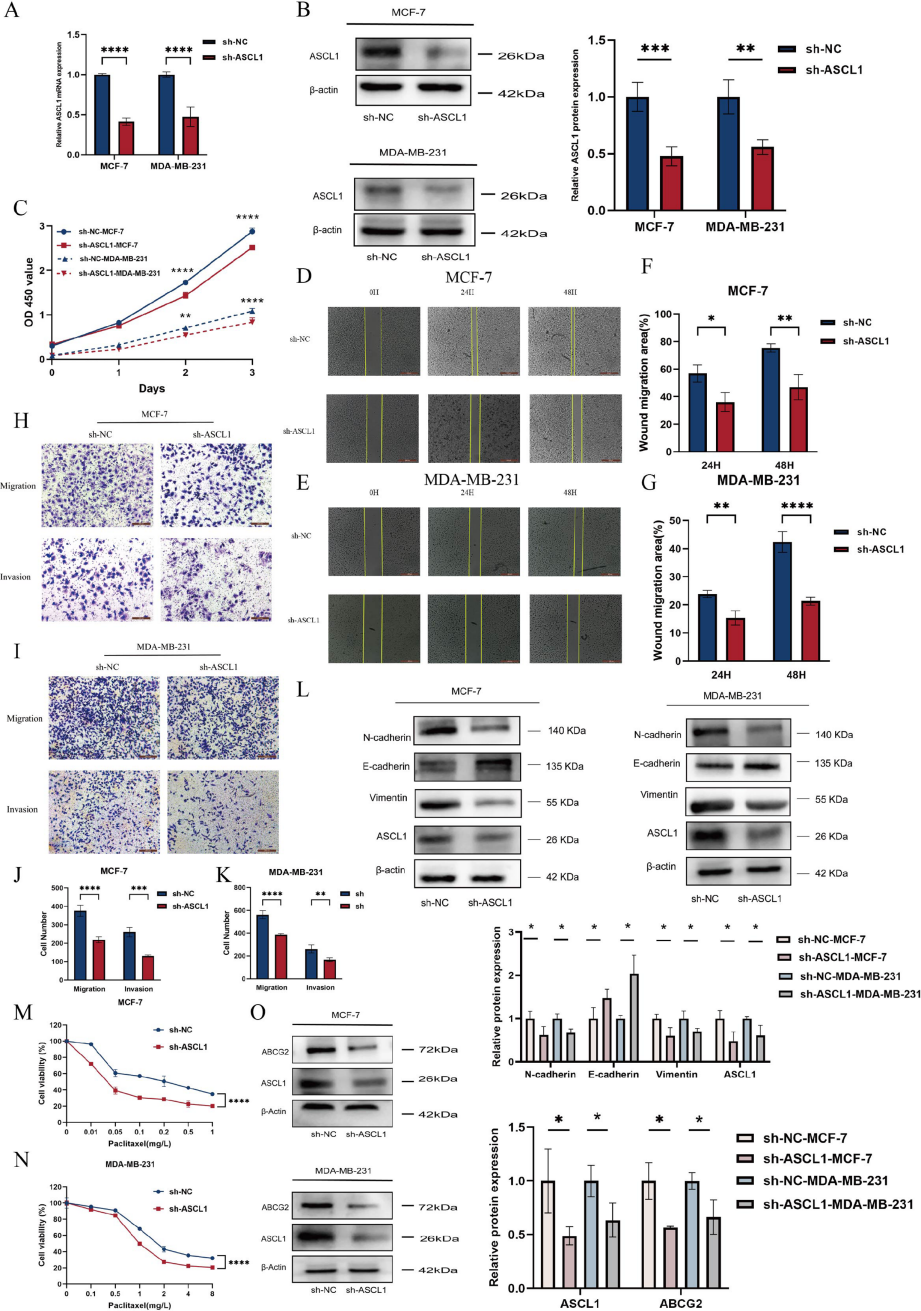
Knockdown of ASCL1 inhibits BC progression *in vitro*. **(A, B)** Evaluation of ASCL1 knockdown efficiency following plasmid transfection using **(A)** qRT-PCR and **(B)** Western blot analysis. **(C)** CCK-8 assay indicates that ASCL1 knockdown suppresses BC cell proliferation *in vitro*. **(D, E)** Wound healing assay showing that ASCL1 knockdown inhibits migration in **(D)** MCF-7 and **(E)** MDA-MB-231 cells (scale bar = 500μm). **(F, G)** Histogram of percentage of wound migration area for **(F)** MCF-7 and **(G)** MDA-MB-231 cells. **(H, I)** Transwell assay demonstrating that ASCL1 knockdown suppresses migration and invasion in **(H)** MCF-7 and **(I)** MDA-MB-231 cells (scale bar = 200μm). **(J, K)** Histogram of the results of transwell assay on **(J)** MCF-7 and **(K)** MDA-MB-231 cells. **(L)** ASCL1 knockdown inhibits the EMT process in BC. **(M, N)** ASCL1 knockdown enhances paclitaxel sensitivity in **(M)** MCF-7 and **(N)** MDA-MB-231 cells. **(O)** Reduction in ABCG2 expression following ASCL1 knockdown in BC cells. (*P < 0.05; **P < 0.01; ***P < 0.001; ****P < 0.0001).

To investigate the potential of targeting ASCL1 to inhibit BC progression *in vitro*, ASCL1 expression was knocked down in BC cells using a transfected plasmid. Both qRT-PCR and Western blot analyses confirmed a significant reduction in ASCL1 levels (**Figures 6A, B**). ASCL1 inhibition led to a marked decrease in cell proliferation (**Figure 6C**). The wound healing and Transwell assays demonstrated that ASCL1 suppression significantly reduced cell migration and invasion (**Figures 6D-K**). Given the role of EMT in promoting cell migration (30), the relationship between ASCL1 and EMT was examined. Western blot analysis confirmed that ASCL1 inhibition suppressed the EMT process, thereby reducing migration and invasion (**Figure 6L**). More importantly, ASCL1 inhibition led to an enhanced sensitivity to paclitaxel, indicating reduced drug resistance (**Figures 6M, N**). Further analysis showed that ASCL1 knockdown resulted in the downregulation of the ATP-binding cassette transporter (ABCG2), a protein linked to drug resistance in BC, also known as the breast cancer resistance protein (31) (**Figure 6O**). The reduction in ABCG2 expression further supports the increased sensitivity of BC cells to paclitaxel following ASCL1 inhibition. These results collectively indicate that targeting ASCL1 effectively impairs BC progression *in vitro*.

### 3.8 Inhibition of ASCL1 improves BC paclitaxel sensitivity through activation of ferroptosis

**Figure 7:**
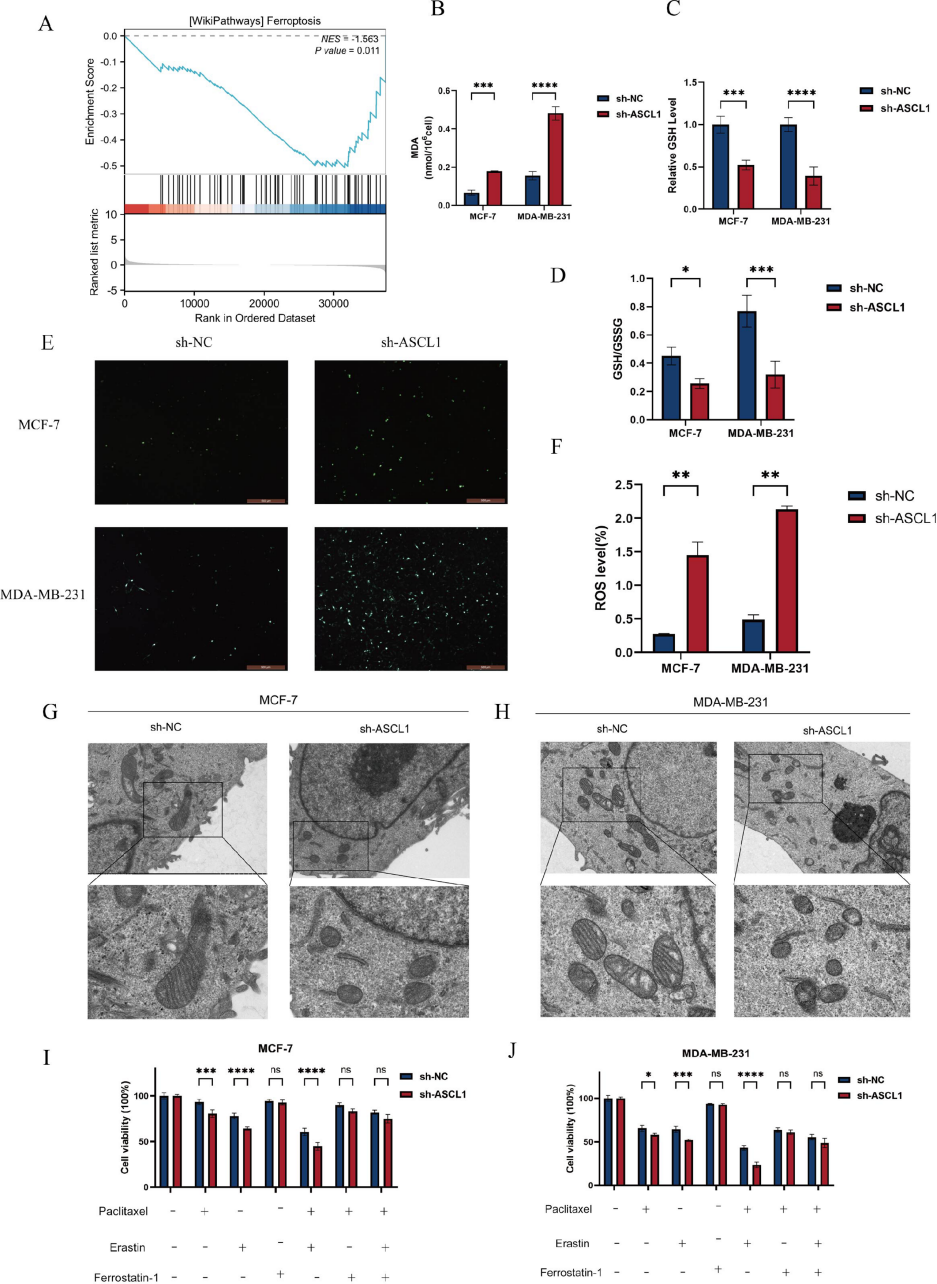
ASCL1 knockdown activates ferroptosis in BC cells. **(A)** GSEA indicates an association between ASCL1 and ferroptosis in BC. **(B)** Increased MDA levels due to ASCL1 knockdown. **(C)** Decreased GSH levels in BC cells with ASCL1 knockdown. **(D)** Reduction in the GSH/GSSG ratio following ASCL1 knockdown. **(E, F)** Elevated ROS levels following ASCL1 knockdown in BC cells (scale bar = 500μm). **(G, H)** TEM showing changes in mitochondrial morphology characteristic of ferroptosis after ASCL1 downregulation in **(G)** MCF-7 and **(H)** MDA-MB-231(scale bar = 2μm and 500 nm). **(I, J)** ASCL1 knockdown enhances paclitaxel sensitivity associated with ferroptosis in **(I)** MCF-7 and **(J)** MDA-MB-231 cells (Erastin: 5 μM, ferrostatin-1: 2 μM, paclitaxel (MCF-7): 0.01 mg/L, paclitaxel (MDA-MB-231): 1 mg/L). (*P < 0.05; **P < 0.01; ***P < 0.001; ****P < 0.0001), ns, not significant.

To further investigate the impact of ASCL1 inhibition on BC cells, we obtained differentially expressed genes (DEGs) from the TCGA-BRCA cohort between the ASCL1 high- and low-expression groups and then performed GSEA analysis. The analysis showed that the DEGs were enriched in the ferroptosis pathway, indicating a potential link between ASCL1 and ferroptosis (**Figure 7A**). To validate this association, intracellular levels of MDA, ROS, GSH, and GSSG were measured, as these are key markers of ferroptosis (32, 33). We found that ASCL1 inhibition resulted in increased levels of MDA and ROS, along with decreased GSH levels and a reduced GSH/GSSG ratio in both sh-ASCL1-MCF-7 and sh-ASCL1-MDA-MB-231 cells (**Figures 7B-F**). Transmission electron microscopy provided further evidence, revealing characteristic features of ferroptosis in the sh-ASCL1 group compared to the sh-NC group, including shrunken mitochondria, reduced size, loss of cristae, and increased membrane density (**Figures 7G, H**) (34). These results collectively confirm that ASCL1 inhibition induces ferroptosis in BC cells.

Given the earlier observation that ASCL1 inhibition enhances paclitaxel sensitivity in BC cells, the potential involvement of ferroptosis was further explored. CCK-8 assays confirmed that sh-ASCL1 BC cells exhibited lower viability compared to sh-NC cells when treated with paclitaxel alone. However, co-treatment with the ferroptosis inhibitor ferrostatin-1 and paclitaxel eliminated the cell viability differences between the two groups. This suggests that ASCL1’s effect on paclitaxel sensitivity is linked to ferroptosis. Additionally, co-treatment with the ferroptosis activator erastin and paclitaxel significantly reduced cell viability, with a more pronounced reduction observed in the sh-ASCL1 group compared to the sh-NC group (**Figures 7I, J**). These results indicate that ASCL1 inhibition may enhance the sensitivity of BC cells to paclitaxel through the activation of ferroptosis.

### 3.9 Inhibition of ASCL1 activates ferroptosis *via* the CREB1/GPX4 axis in BC

**Figure 8:**
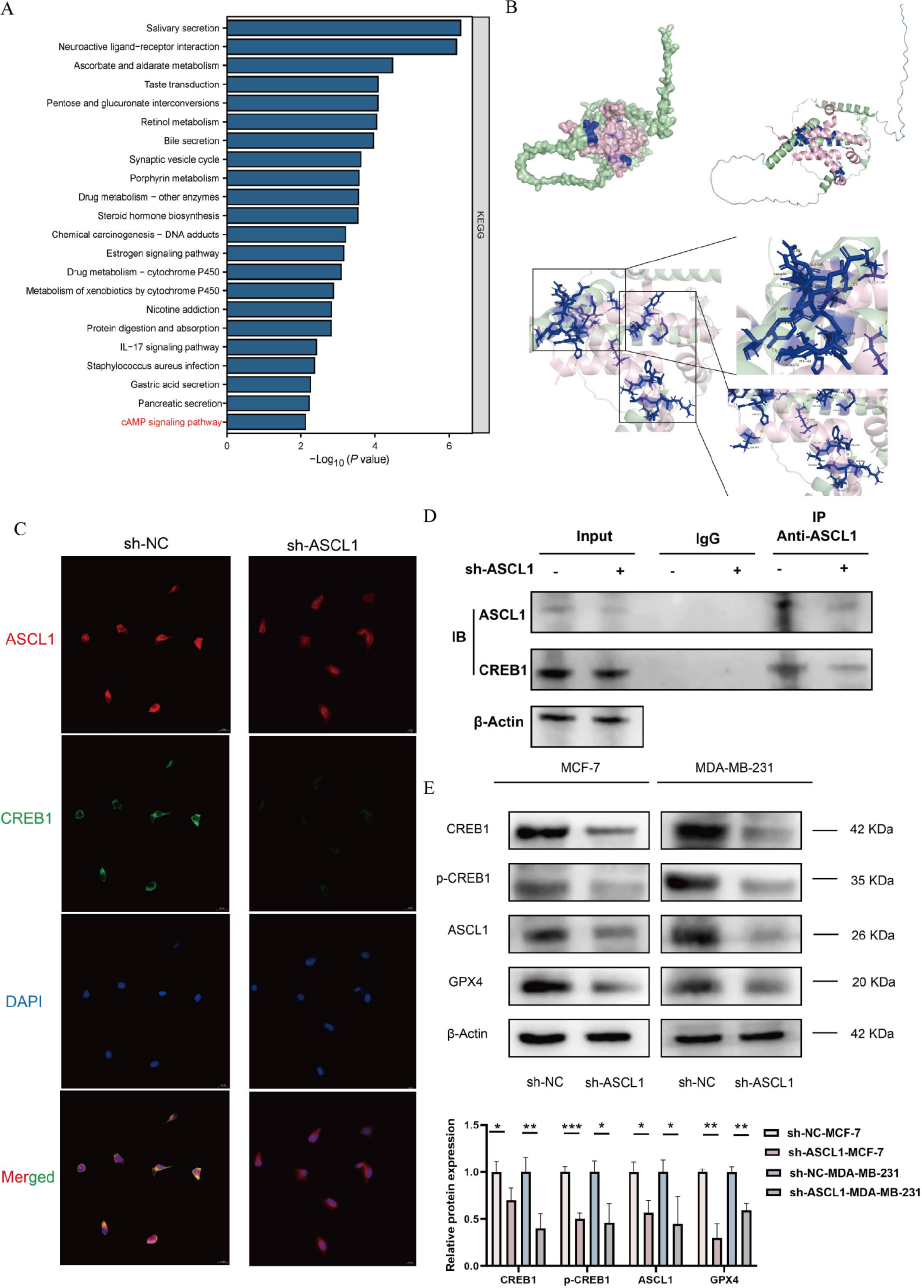
Inhibition of ASCL1 activates ferroptosis *via* the CREB1/GPX4 axis. **(A)** KEGG analysis indicates an association between ASCL1 and the cAMP pathway. **(B)** Protein docking map and binding site of ASCL1 to CREB1. **(C)** Immunofluorescence assay illustrates co-localization of ASCL1 with CREB1. **(D)** Co-IP assay indicates that ASCL1 binds to CREB1 in BC. **(E)** ASCL1 knockdown inhibits the CREB1/GPX4 pathway in BC cells. (*P < 0.05; **P < 0.01; ***P < 0.001).

To elucidate the underlying mechanisms, 474 DEGs, comprising 248 upregulated and 226 downregulated genes, were identified between high- and low-ASCL1 expression groups from the TCGA-BRCA cohort using a cutoff of |log2FC| > 1 and adjusted *P* < 0.05 for KEGG pathway analysis. The results indicated an association between ASCL1 and the cAMP signaling pathway (**Figure 8A**). Through bioinformatics analysis, we finally identified cAMP-responsive element-binding protein 1 (CREB1) as the target gene of ASCL1 for regulating ferroptosis in BC (**Supplementary Figure 1**). CREB1 is a phosphorylation-dependent transcription factor. It regulates the transcription of GPX4, a key inhibitor of ferroptosis (**Supplementary Figure 1**). Thus, the mechanism by which ASCL1 regulates ferroptosis in BC may be related to the CREB1/GPX4 axis.

To verify our hypothesis, we first performed protein docking on the binding of ASCL1 to CREB1. **Figure 8B** displays the structures and binding sites of the two proteins. The green one is ASCL1, the pink one is CREB1, and the blue part is the binding site of the two proteins. It was found that there was a very tight hydrogen bond connection between the two proteins, suggesting that there may be a potential association between ASCL1 and CREB1. We then implemented immunofluorescence experiments and found that ASCL1 and CREB1 co-localized in BC cells and that inhibition of ASCL1 significantly suppressed the co-localization (**Figure 8C**). The Co-IP experiments also revealed that ASCL1 was bound to CREB1 in BC and that inhibition of ASCL1 promotes complex dissociation (**Figure 8D**). These results suggest a possible regulatory association between ASCL1 and CREB1. Western blot analysis further confirmed that inhibition of ASCL1 in BC cells reduced the phosphorylation of CREB1 at Ser133 (**Figure 8E**). CREB1 is a phosphorylation-dependent transcription factor whose activity is significantly boosted upon phosphorylation (35). Reduced phosphorylation of CREB1 resulted in decreased expression of GPX4, leading to activation of ferroptosis (**Figure 8G**). In summary, this study confirmed that ASCL1 regulates ferroptosis through the CREB1/GPX4 axis in BC.

### 3.10 Inhibition of ASCL1 increases BC sensitivity to paclitaxel *in vivo*

**Figure 9:**
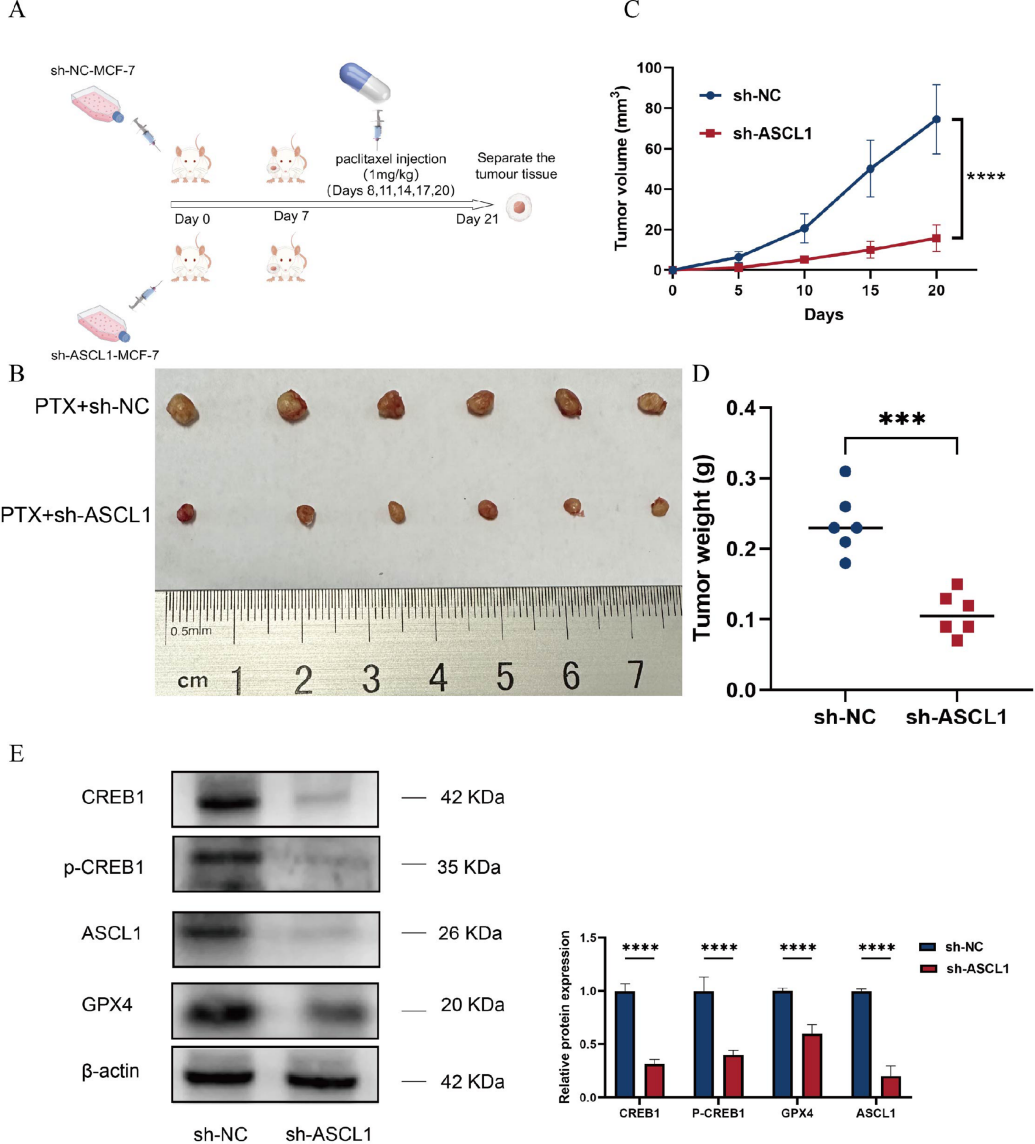
ASCL1 knockdown enhances BC sensitivity to paclitaxel *in vivo*. **(A)** Schematic representation of the *in vivo* experimental design. **(B)** Photograph of tumors collected at the end of the experiment. **(C)** Tumor volume progression over time in both groups during the experiment. **(D)** Comparison of tumor weights between the two groups at the conclusion of the experiment. **(E)** ASCL1 knockdown inhibits the CREB1/GPX4 pathway *in vivo*. (***P < 0.001; ****P < 0.0001).

The cytological experiments demonstrated that ASCL1 inhibition enhances paclitaxel sensitization *in vitro*. To confirm these effects *in vivo*, experiments were conducted using female BALB/c nude mice, following the protocol outlined in **Figure 9A**. The results showed significantly lower tumor volume and weight in the sh-ASCL1 group compared to the sh-NC group (**Figures 9B-D**). Notably, western blot analysis further demonstrated that knockdown of ASCL1 inhibited the CREB1/GPX4 pathway *in vivo* (**Figure 9E**). These results were consistent with those of *in vitro* experiments, jointly demonstrating that targeting ASCL1 could inhibit BC progression and increase BC sensitivity to paclitaxel by inducing ferroptosis.

## 4 Discussion

Previous studies have implicated ASCL1 in the initiation and progression of various cancers, where it promotes tumor cell proliferation (11–13), supports cell survival and differentiation (36), and influences drug sensitivity (37). However, its role in BC remains unclear. In this study, we elucidated the function and regulatory mechanism of ASCL1 in BC through comprehensive bioinformatics analysis, cytological experiments and animal experiments.

Our bioinformatics analysis revealed that ASCL1 expression is significantly elevated in BC and associated with poor prognosis. Additionally, ASCL1 mutation frequency was notably higher in metastatic BC compared to primary tumors, and its expression correlated with mutation rates in key BC driver genes, such as TP53 and PIK3R1. The study further explored the relationship between ASCL1 expression and various immunomodulatory factors, including cytokines, immunoregulators, MHC molecules, and receptors. Given the established role of immunomodulatory factors in shaping the tumor microenvironment and influencing immune responses (26, 38, 39), our findings of a negative correlation between ASCL1 levels and these factors suggest an immunosuppressive effect in BC. Notably, ASCL1 expression was inversely associated with CXCL10 and CCL20, consistent with previous research (13), which showed that ASCL1 downregulates these chemokines, impairing immune cell migration and contributing to an “immune desert” phenotype, thus compromising immune responses in cancers such as lung adenocarcinoma (13). The analysis also showed a link between ASCL1 expression and immune cell infiltration as well as immunoreactivity scores, which also demonstrates a possible association of ASCL1 with the immune response to BC.

*In vitro* experiments corroborated the bioinformatics findings, demonstrating that ASCL1 overexpression accelerates BC progression by enhancing cell proliferation, migration, and invasion, whereas ASCL1 inhibition yields the opposite effects. Importantly, the inhibition of ASCL1 significantly increased paclitaxel sensitivity both *in vitro* and *in vivo*, aligning with the bioinformatics data and suggesting that targeting ASCL1 could improve therapeutic outcomes in BC.

Enrichment analysis revealed a link between ASCL1 and ferroptosis, a distinct form of cell death characterized by iron-dependent lipid peroxidation, differing from apoptosis and necrosis (40). Emerging evidence suggests that ASCL1 can protect cells from oxidative stress, thereby promoting differentiation (41). Since oxidative stress and lipid peroxidation are key precursors to ferroptosis (42), this study further investigated the role of ASCL1 in this pathway. Knockdown of ASCL1 increased intracellular levels of ROS and MDA, specific markers of lipid peroxidation associated with ferroptosis (33). Additionally, transmission electron microscopy showed mitochondrial changes characteristic of ferroptosis following ASCL1 inhibition, indicating that ASCL1 may be involved in the key steps of the ferroptosis process in BC. Inhibition of ASCL1 significantly activated ferroptosis in BC cells.

Previous research has demonstrated the therapeutic potential of inducing ferroptosis in cancer treatment, including overcoming drug resistance (43). For instance, in hepatocellular carcinoma, Nrf2 pathway activation confers resistance to sorafenib by inhibiting ferroptosis (44). Similarly, in cisplatin-resistant head and neck cancers, inducing ferroptosis reverses resistance (45). Consistent with these results, this study showed that ASCL1 inhibition enhances BC sensitivity to paclitaxel, while the use of a ferroptosis inhibitor abolished this effect. Conversely, applying ferroptosis inducers further amplified the paclitaxel sensitivity, suggesting that ASCL1 inhibition augments paclitaxel efficacy through ferroptosis activation in BC.

Mechanistic studies were conducted to elucidate the underlying processes. GSH, catalyzed by GPX4, scavenges intracellular ROS, providing a defense against oxidative stress-induced cell death. The GSH/GPX4 pathway serves as a key mechanism for inhibiting ferroptosis. Findings from this study revealed that there is an interaction between ASCL1 and CREB1 in BC and that GPX4 is a downstream target of the transcription factor CREB1. Inhibition of ASCL1 reduces the phosphorylation of CREB1, which in turn decreases the expression of GPX4 and consequently activates ferroptosis.

Our study initially revealed that targeting ASCL1 activates ferroptosis in BC via the CREB1/GPX4 axis thereby increasing BC sensitivity to paclitaxel and inhibiting BC progression. These results are expected to provide new insights into the treatment of BC. However, the cytological studies in this study only investigated the effects of ASCL1 on proliferation, migration, invasion, and paclitaxel sensitivity in BC, and further studies are still needed to clarify the other roles of ASCL1 in BC and its potential as a therapeutic target. In addition, this study only preliminarily revealed the effect of ASCL1 on ferroptosis, and it remains to be investigated the mechanism in more depth in the future. These are the limitations of this study.

## 5 Conclusions

This study demonstrated that ASCL1 is upregulated in BC and correlates with poor prognosis. ASCL1 plays multiple oncogenic roles in BC, influencing tumor immunity, drug sensitivity, and cellular behaviors such as proliferation, migration, invasion, and EMT. While ASCL1 overexpression promotes these malignant processes, its knockdown produces the opposite effects. Notably, ASCL1 inhibition enhances BC sensitivity to paclitaxel both *in vitro* and *in vivo* by activating ferroptosis via the CREB1/GPX4 axis. These findings suggest that ASCL1 could serve as a promising prognostic biomarker and therapeutic target for patients with BC.

## Data Availability

The original contributions presented in the study are included in the article/**Supplementary Material**. Further inquiries can be directed to the corresponding author/s.
