# Supplementary material for: An integrative analysis of ASCL1 in breast cancer and inhibition of ASCL1 increases paclitaxel sensitivity by activating ferroptosis via the CREB1/GPX4 axis
Source: Front Immunol. 2025 Feb 3;16:1546794. doi: 10.3389/fimmu.2025.1546794 (PMC11830715; doi:10.3389/fimmu.2025.1546794)
Supplement: Supplementary file 1 [file DataSheet1.docx]

## Supplementary Figure 1


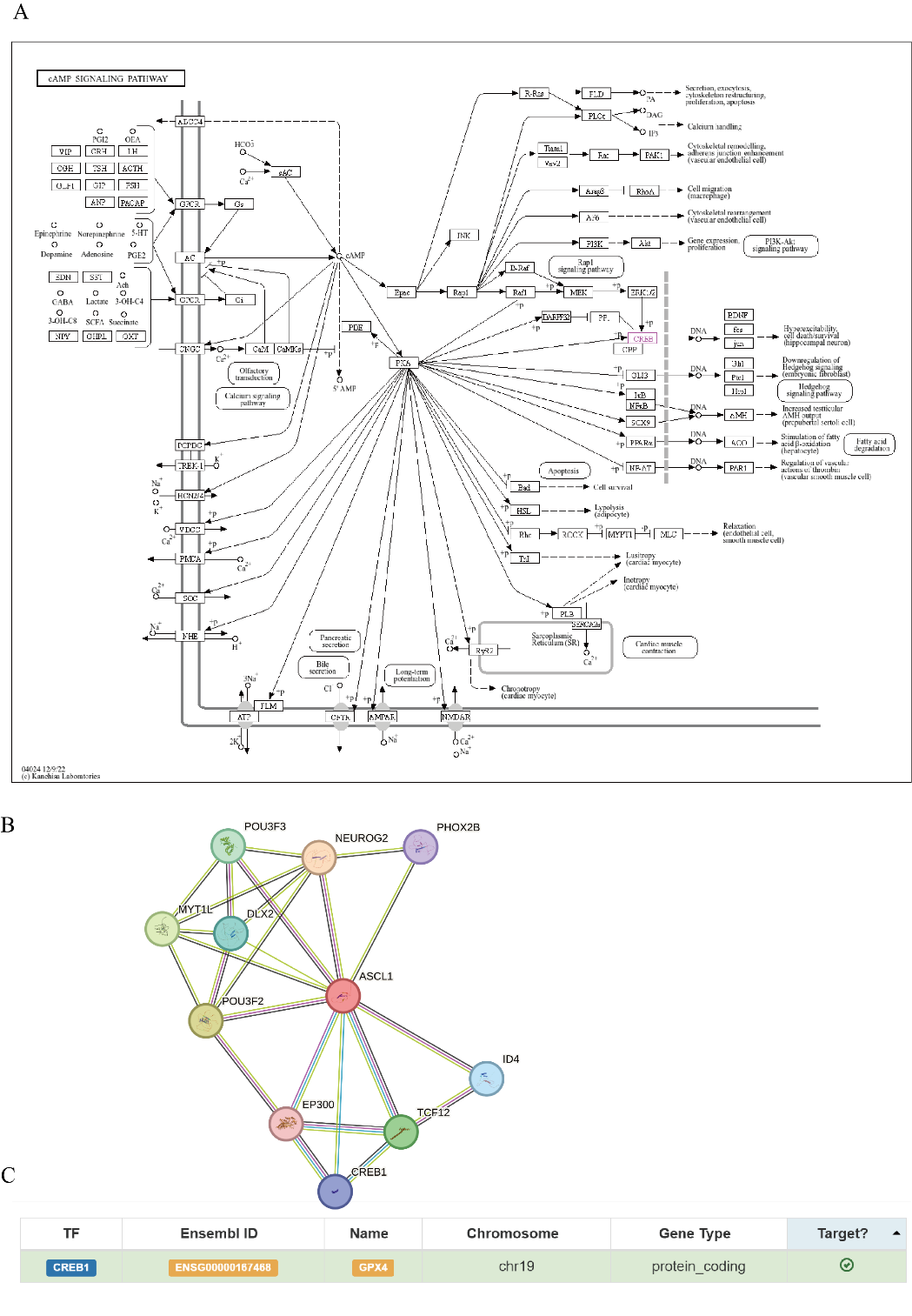


**Supplementary Figure 1.** ASCL1 regulates ferroptosis through the CREB1/GPX4 axis. (A) Annotation of cAMP signalling pathway. (B) Proteins that interact with ASCL1. (C) GPX4 is a target gene of the transcription factor CREB1.
